# Association Between Social Networking Site Use Intensity and Depression Among Chinese Pregnant Women: Cross-sectional Study

**DOI:** 10.2196/41793

**Published:** 2023-03-15

**Authors:** Rui Wang, Shengnan Cong, Lijuan Sha, Xiaoqing Sun, Rong Zhu, Jingyi Feng, Jianfang Wang, Xiaomei Tang, Dan Zhao, Qing Zhu, Xuemei Fan, Ziqi Ren, Aixia Zhang

**Affiliations:** 1 School of Nursing Nanjing Medical University Nanjing China; 2 Department of Gynecology Women’s Hospital of Nanjing Medical University, Nanjing Maternity and Child Health Care Hospital Nanjing China; 3 Department of Obstetrics Suqian First Hospital Suqian China; 4 Department of Obstetrics Women’s Hospital of Nanjing Medical University, Nanjing Maternity and Child Health Care Hospital Nanjing China; 5 Department of Applied Biology and Chemical Technology Faculty of Science The Hong Kong Polytechnic University Hongkong China; 6 Department of Nursing Changzhou Maternal and Child Health Care Hospital Changzhou China; 7 Department of Nursing Xinghua Maternity and Child Healthcare Centre Taizhou China; 8 Department of Obstetrics Changzhou Maternal and Child Health Care Hospital Changzhou China; 9 School of Nursing Fudan University Shanghai China; 10 Department of Nursing Women’s Hospital of Nanjing Medical University Nanjing Maternity and Child Health Care Hospital Nanjing China

**Keywords:** antenatal depression, social network site, social media, WeChat, upward social comparison, rumination

## Abstract

**Background:**

Despite extensive debates about the mental health impacts of the use of social networking sites (SNSs), including WeChat, the association and mechanisms between social interaction of WeChat use intensity and antenatal depression are unclear.

**Objective:**

We aimed to test the mediating roles of upward social comparison on social interaction of WeChat and rumination in the association between social interaction of WeChat use intensity and antenatal depression.

**Methods:**

A cross-sectional survey was conducted in four hospitals with the self-reported measures of social interaction of WeChat use intensity, upward social comparison on social interaction of WeChat, rumination, antenatal depression, and control variables. The mediation analysis was performed through Model 6 from the PROCESS macro 4.0 in SPSS 26.

**Results:**

Results from 2661 participants showed that antenatal depression was unrelated to social interaction of WeChat use intensity (*P*=.54), but was significantly positively related to the attitude toward social interaction of WeChat (*P*=.01). The direct effect of attitude toward social interaction of WeChat use on antenatal depression was not statistically significant (β=–.03, *P*=.05). The results supported an indirect relationship between attitude toward social interaction of WeChat use and antenatal depression via (1) upward social comparison on social interaction of WeChat (indirect effect value=0.04, 95% CI 0.03 to 0.06); (2) rumination (indirect effect value=–0.02, 95% CI –0.04 to –0.01); and (3) upward social comparison on social interaction of WeChat and rumination in sequence (indirect effect value=0.07, 95% CI 0.06 to 0.08).

**Conclusions:**

Our findings highlight the necessity of focusing on attitudes toward SNS use, and the importance of upward social comparison and rumination in understanding the effect of SNS use on antenatal depression.

## Introduction

Antenatal depression is typically defined as a nonpsychotic depressive episode of mild to major severity that occurs during pregnancy [1]. A meta-analysis revealed that the prevalence of antenatal depression is 25.5%, which is higher than the prevalence of postpartum depression (19.6%) [1]. Furthermore, the far-reaching influences of antenatal depression cannot be ignored from the scale of mothers and their offspring to society as a whole. First, women with antenatal depression suffer from inability to feel pleasure, changes in appetite and sleep, psychomotor retardation, poor concentration [2], and even suicide [3]. Second, antenatal depression is associated with increased risks of preterm birth [4] and low birth weight [4], and even cause poorer offspring social-emotional, cognitive, language, motor, and adaptability outcomes through infancy, childhood, and adolescence [5]. Third, the aggregated value of lifetime costs per woman for antenatal depression was estimated at US $55,143 [6].

Considering the high prevalence and adverse effects of antenatal depression, it is necessary to explore the comprehensive influencing factors underlying the condition, which is conducive to early detection and further treatment of groups at higher risk of antenatal depression. A recent meta-analysis revealed that the main factors associated with antenatal depression include pregnant women’s demographic characteristics, psychosocial factors, health-related factors, lifestyle, and nutrition [7]; however, previous studies have largely ignored the potential effects of social networking sites (SNSs) use.

Since the concept of “Facebook depression” was first proposed, which is a term to simply describe the positive association between time spent on SNSs and depression [8], the effects of SNS use on depression have been among the hottest debates in academia and society at large. With further research, the intensity of SNS use has been put forward as an important variable mediating depression, which includes three aspects: time spent on SNS, number of SNS friends, and attitudes toward SNS (ie, the extent to which individuals are emotionally connected to SNSs and the extent to which SNS use is integrated into their daily activities) [9]. A recent umbrella review demonstrated that the reported associations of intensity of SNS use and depression are inconsistent [10], which highlights the need to further explore the mechanisms linking these factors.

Toward this end, our research focused on investigating the relationship between the intensity of SNS use and antenatal depression, with the aim to further explore the underlying mechanisms from a new perspective by examining the mediating roles of upward social comparison and rumination.

Upward social comparison refers to the process of comparing oneself to others who are considered to be superior [11]. Upward social comparison exists across cultures [12] and has been raised as a core feature of human social evolution [13]. This further provides opportunities to perform self-assessment [14], enabling navigating the social world successfully. Therefore, upward social comparisons are ubiquitous and difficult to resist. Furthermore, greater intensity of SNS use evokes upward social comparisons [15,16]. This is likely due to SNSs making it easier for individuals to portray idealized images, so that more intense SNS use inevitably exposes individuals to others’ positively skewed self-presentations [17]. Therefore, the intensity of SNS use is positively associated with higher tendency for upward social comparison.

A recent meta-analysis demonstrated that upward social comparison on SNSs is associated with higher risks of depression, which even exceeds the association for SNS use itself [18]. This finding can be explained by social rank theory [19], which proposes that after upward social comparison, involuntary responses such as submissiveness, withdrawal, and self-criticism are yielded, which are indicative of depression. Based on this, we hypothesized that upward social comparison on SNSs is an independent mediator between the intensity of SNS use and antenatal depression.

However, given that the key factor explaining the causal link between upward social comparison and depression is unclear, at this point, we can only recommend users to abstain from upward social comparisons while on SNSs. Such a recommendation seems futile, because experiencing upward social comparison on SNSs is an inherent part of the human condition. Thus, it is necessary to explore the mechanisms underlying the relationships between upward social comparison on SNSs and depression, including rumination.

Rumination is defined as repetitive thinking about the causes and implications of negative events and/or the symptoms of negative moods [20]. Conceptual models regarding the etiology of rumination suggest that a stressful event is the antecedent of rumination [21,22], and upward social comparison is one of the most frequent SNS stressors [23]. Moreover, upward social comparison usually invites negative moods such as envy and lower self-esteem [24-26]. The attentional scope model provides a cognitive explanation for why individuals are more likely to ruminate when in negative mood states [27]. This model postulates that negative moods lead to individuals’ narrowing scope of attentional selection, which in turn causes more stable maintenance of negative moods and difficulties getting away or disengaging from those moods, thus resulting in persistent repetitive thinking about negative moods (ie, rumination). Therefore, upward social comparison provides the perfect conditions for creating rumination.

Notably, a wealth of evidence demonstrates that rumination is a risk and maintenance factor of depression. The Response Styles Theory provides explanations for the mechanisms between rumination and depression [20,28]. First, rumination amplifies a bidirectional circuit between negative moods and negative thinking, thereby increasing the availability of negative information when individuals are in negative moods and increasing their affective reactivity to adverse information. Second, rumination leads to individuals thinking more pessimistically and fatalistically, thus hindering problem-solving. Third, rumination interferes with individuals’ instrumental behaviors. Finally, rumination reduces social support by creating unwelcome interpersonal characteristics (eg, excessive neediness), consequently repelling potential help mates.

In addition, research demonstrates differences among the effects of different types of SNSs on individuals; therefore, it is recommended that researchers should clearly specify which SNS is being investigated [25,29,30]. Given that our study was conducted in the Chinese context, we selected to perform the present analysis based on the WeChat platform, which has the highest number of users in China (1263 million) [31]. WeChat is equivalent to other SNSs in Western countries (such as Facebook and Twitter) with respect to its function, which allows social interactions in “Moments” through posting something, browsing others’ posts, making comments, and so on, along with instant messaging (chatting with friends), gaming, paying, or shopping. WeChat also has its own distinctive features, as this platform is based on realistic relationships and is therefore considered to be relatively more private than Facebook and Twitter. Our study focused on the function of social interaction because numerous posts in Moments are the main triggers of upward social comparison. Therefore, we refer to intensity of SNS use as the social interaction of WeChat use intensity for this study.

**Figure 1:**
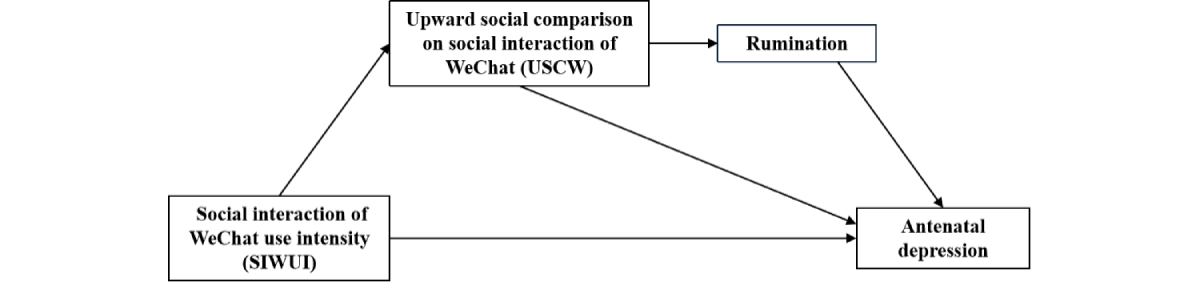
Hypothesized conceptual model of the chain mediation between upward social comparison on social interaction of WeChat and antenatal depression.

We hypothesized that: (1) upward social comparison on social interactions of WeChat is an independent mediator, (2) and upward social comparison on social interaction of WeChat and rumination are the chain mediators linking social interaction of WeChat use intensity and antenatal depression (see Figure 1).

## Methods

### Study Design, Procedure, and Population

Cross-sectional data were collected from October 2021 to January 2022 using Wenjuanxing (an online data collection platform) through a convenience sampling method. To control the effect of missing data, the questionnaire was designed to ensure that valid respondents need to complete all items before submitting using the features of Wenjuanxing. Therefore, there was no need to apply methods for handling missing data in our study.

We recruited pregnant women from four hospitals in Jiangsu Province, China. Inclusion criteria were (1) having basic understanding and communication skills and (2) voluntary participation. Exclusion criteria were (1) suffering from or with a history of mental illness, (2) receiving psychotherapy or a psychological intervention, and (3) not users of WeChat.

### Outcome Measures

Participants completed the measures, including assessments of social interaction of WeChat use intensity as the independent variable, upward social comparison on social interaction of WeChat and rumination as the two chain mediating variables, antenatal depression as the dependent variable, and control variables. Multimedia Appendix 1 provides all the measurement items.

We assessed the social interaction of WeChat use intensity (independent variable) with the Facebook intensity scale [9]. We changed “Facebook” into social interaction of WeChat. The measure included two items to measure time spent on social interaction of WeChat use per day (0=less than 10 minutes, 1=10-30 minutes, 2=31-60 minutes, 3=1-2 hours, 4=2-3 hours, 5=more than 3 hours) and number of WeChat friends (0=10 or less, 1=11-50, 2=51-100, 3=101-150, 4=151-200, 5=201-250, 6=251-300, 7=301-400, 8=more than 400), and six items to measure the attitude toward social interaction of WeChat use with a 5-point scale (1=strongly disagree to 5=strongly agree).

Upward social comparison on social interaction of WeChat (mediator 1) was measured with the six-item Chinese version of the upward comparison subscale of Iowa-Netherlands Comparison Orientation Measure (1=strong disagreement, 5=strong agreement) [32]. We shifted the scope of comparison to social interaction of WeChat use on the existing scale. Higher scores indicated higher frequencies of upward social comparison on social interaction of WeChat.

Rumination (mediator 2) was measured by the Chinese version of the 22-item Ruminative Response Scale with a 4-point Likert scale (1=not at all, 4=very often) [33]. Higher scores meant higher frequencies of rumination in response to negative events.

Antenatal depression (dependent variable) was measured by the Chinese version of the 10-item Edinburgh Postnatal Depression Scale (EPDS) with each item scored on a scale of 0-3 [34]. This scale has already been validated for use in screening antenatal depression [35-38]. Higher scores reflect greater levels of self-reported depressive symptoms; a total score above 9.5 represents a probable risk of antenatal depression.

To reduce other possible influences on our model, we considered control variables, which were not the variables of primary interest for this study. To reduce the possible influence of individual differences, demographic information such as education level (below junior college=1, junior college=2, bachelor degree=3, master degree or above=4), job (middle level and above management personnel=1, professional and technical personnel=2, clerks and relevant personnel=3, social production service and life service personnel=4, production and manufacturing personnel and related personnel=5, other unclassifiable jobs=6, have no job=7), advanced maternal age or not (yes=1, no=2), and family income (1=<US $287.60, 2=US $287.60-575.20, 3=US $575.30-862.80, 4=US $862.90-1150.40, 5=US $1150.40-1438.10, 6=US $1438.10-2876.00, 7=>US $2876.00) were considered. To reduce the possible influence of the respondents’ characteristics related to antenatal depression, we also controlled for psychosocial and obstetric variables such as quality of relationship with husband and mother-in-law (very good=1, good=2, general=3, poor=4, terrible=5), whether it is an unplanned pregnancy (yes=1, no=2), living conditions (very good=1, good=2, general=3, poor=4, terrible=5), the main caregivers during pregnancy (mother-in-law=1, mother=2, husband=3, nursemaid=4, others=5), gestational age (early pregnancy=1, second trimester=2, late pregnancy=3), gestation (0=none, 1=once, 2=twice, 3=three times or above), parturition (0=none, 1=once, 2=twice or above), BMI (<18.5=1, 18.5-23.9=2, ≥24=3, ≥28=4), and experiences of abnormal pregnancies and labor (yes=1, no=2).

### Ethical Considerations

Ethics approval was granted by the Ethics Committee of Nanjing Maternity and Child Health Care Hospital (approval number 2021KY-086). All procedures conducted in our study were in adherence with the ethical guidelines of the institutional and/or national research committee and the ethical standards of the Helsinki Declaration. Informed consent was obtained from all participants included in the study.

### Common Method Variance

As with all self-reported data, it is important to control and examine the potential common method variance. First, we took actions to minimize potential common method biases [39]. To reduce respondents’ evaluation apprehension consideration for social desirability, they were reminded that their answers were anonymous and there were no “right” or “wrong” answers to our questions. To solve potential ambiguity in wording, a pretest with 20 participants was conducted. Based on the pretest results, the final version of the questionnaire was developed after modifications. The Harman single-factor test was performed to determine whether common method bias is a problem [40]. The assumption of this test is that if substantial common method variance exists, either (1) a single factor emerged or (2) one general factor (such as the first factor) accounted for the majority of the covariance (>50%) among the measures.

### Data Analysis

#### Data Analytical Tool

Data analysis was carried out in IBM SPSS Statistics (version 26). Model 6 in the PROCESS macro for SPSS was used to examine the mediating roles of upward social comparison on social interaction of WeChat and rumination.

#### Data Processing

We processed the data on the social interaction of WeChat use intensity as follows. First, we evaluated three independent aspects: time spent on social interactions of WeChat, number of WeChat friends, and attitude toward social interaction of WeChat use. Due to different item scale ranges, the eight items were standardized before creating the total scores of social interaction of WeChat use intensity. Therefore, a total of four aspects of WeChat use were considered.

#### Descriptive Statistics

Descriptive statistics were calculated to summarize the characteristics of each variable, including mean, standard deviation, skewness, and kurtosis. Normality was considered adequate if absolute values for skewness and kurtosis were below 3.0 and 10.0, respectively [41,42].

#### Reliability Analysis

We used Cronbach α to represent the internal consistency reliability. A Cronbach α over .70 is considered an indicator of satisfactory item homogeneity.

#### Mediation Analysis

First, we calculated Pearson correlation coefficients between social interactions of WeChat use, number of WeChat friends, attitude toward social interaction of WeChat use, social interaction of WeChat use intensity, upward social comparison on social interaction of WeChat, rumination, and antenatal depression. We then performed an independent *t*-test (2-tailed) or ANOVA to find the control variables that are significantly correlated with antenatal depression, which needed to be controlled as covariates in the following mediation analysis. The standardized coefficients and 95% CIs for direct, indirect, and total effects were calculated through the bias-corrected bootstrap approach (5000 resamples). If the 95% CI did not contain 0, this indicated statistical significance. Two-tailed *P* values <.05 were considered to be significant.

The STROBE (Strengthening The Reporting of Observational Studies in Epidemiology) checklist for cross-sectional studies was applied in our study (see Multimedia Appendix 2).

## Results

### Common Method Biases

There was no single factor emerging from the factor analysis and the variation explained by the first factor was 21.1% (<50%), demonstrating that there was no serious risk of common method bias in our study [43].

### Descriptive Statistics and Correlations

Of the 2661 valid questionnaires included in the analysis, 689 women had an EPDS score above 9.5, indicating that 25.9% of our sample may have antenatal depression. Table 1 shows the descriptive statistics, including means (SDs) and Cronbach α values for the main variables. All of the absolute values for skewness and kurtosis were below 3.0 and 10.0, respectively, indicating that the data obeyed a normal distribution.

Table 2 presents the correlations among the key variables. Attitude toward social interaction of WeChat use, upward social comparison on social interaction of WeChat, rumination, and antenatal depression were significantly related to each other. Number of WeChat friends was significantly negatively related to antenatal depression. However, neither social interaction of WeChat use intensity nor time spent on WeChat was related to antenatal depression.

**Table 1:** Descriptive statistics summary (N=2661).

Variable	Mean (SD)	Skewness (SE)	Kurtosis (SE)	Cronbach α
Number of WeChat friends	5.4 (2.32)	–0.36 (0.05)	–1.19 (0.10)	—^a^
Hours spent on social interaction of WeChat use	2.65 (1.72)	0.07 (0.05)	–1.32 (0.10)	—
Attitude toward social interaction of WeChat use	16.77 (5.02)	–0.11 (0.05)	–0.50 (0.10)	—
SIWUI^b^	0.00 (0.26)	–1.18 (0.05)	–0.45 (0.10)	.80
USCW^c^	13.65 (5.04)	0.21 (0.05)	–0.66 (0.10)	.90
Rumination	36.13 (8.36)	0.35 (0.05)	0.68 (0.10)	.94
Antenatal depression	7.72 (3.34)	0.56 (0.05)	1.63 (0.10)	.81

^a^Not applicable.

^b^SIWUI: social interaction of WeChat use intensity; each item was first standardized before taking an average to create the scale due to different item scale ranges.

^c^USCW: upward social comparison on social interaction of WeChat.

**Table 2:** Correlation analysis (Pearson correlation coefficients and two-tailed *P* values) among the key variables (N=2661).

Variable			Number of WeChat friends		Hours spent on social interaction of WeChat use		Attitude toward social interaction of WeChat use		SIWUI^a^		USCW^b^		Rumination		Antenatal depression
**Number of WeChat friends**															
	*r*	1		0.22		0.15		0.65		0.08		0.00		–0.06	
	*P* value	—^c^		<.001		<.001		<.001		<.001		.70		.002	
**Hours spent on social interaction of WeChat**															
	*r*	0.22		1		0.32		0.73		0.12		–0.01		0.04	
	*P* value	<.001		—		<.001		<.001		<.001		.66		.05	
**Attitude toward social interaction of WeChat use**															
	*r*	0.15		0.32		1		0.70		0.37		0.09		0.05	
	*P* value	<.001		<.001		—		<.001		<.001		<.001		.01	
**SIWUI**															
	*r*	0.65		0.73		0.70		1		0.28		0.04		0.01	
	*P* value	<.001		<.001		<.001		—		<.001		.04		.54	
**USCW**															
	*r*	0.08		0.12		0.37		0.28		1		0.39		0.31	
	*P* value	<.001		<.001		<.001		<.001		—		<.001		<.001	
**Rumination**															
	*r*	0.00		–0.01		0.09		0.04		0.39		1		0.60	
	*P* value	.70		.66		<.001		.04		<.001		—		<.001	
**Antenatal depression**															
	*r*	–0.06		0.04		0.05		0.01		0.31		0.60		1	
	*P* value	.002		.05		.01		.54		<.001		<.001		—	

^a^SIWUI: social interaction on WeChat use intensity; each item was first standardized before taking an average to create scale due to different item scale ranges.

^b^USCW: upward social comparison on social interaction of WeChat.

^c^Not applicable.

### Covariates

Among all of the considered control variables, gestational age, education level, gravidity, parity, family income, quality of relationship with husband and mother-in-law, whether it is an unplanned pregnancy, living conditions, and job were significantly correlated with antenatal depression, and were therefore set as covariates.

### Mediating Effect Analysis

Attitude toward social interaction of WeChat use, upward social comparison on social interaction of WeChat, rumination, and antenatal depression were significantly correlated with each other, which met the statistical requirements for mediation analysis.

**Figure 2:**
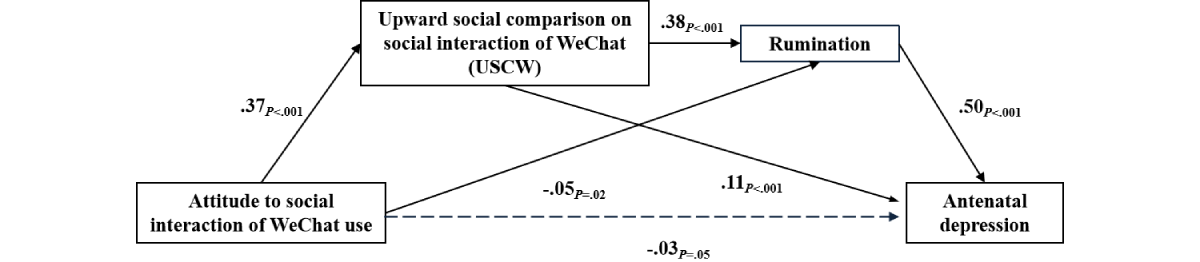
The chain mediating effect of upward social comparison on social interaction of WeChat (USCW) and rumination (N=2661). Values on paths are path coefficients (standardized beta coefficients).

Table 3 shows the detailed outcomes for our proposed mediation model, including model summary, estimates, 2-tailed *t* test, and *P* values for the linear regressions. Figure 2 shows the mediation model, including estimates and *P* values for the linear regressions. The results showed that the direct effect of attitude toward social interaction of WeChat use on antenatal depression was not statistically significant (β=–.03, *P*=.05), whereas attitude toward social interaction of WeChat use significantly predicted upward social comparison on social interaction of WeChat (β=.38, *P*<.001) and rumination (β=–.05, *P*=.02). Upward social comparison on social interaction of WeChat significantly predicted rumination (β=.38, *P*<.001) and antenatal depression (β=.11, *P*<.001). In addition, rumination significantly predicted antenatal depression (β=.50, *P*<.001). These results indicated that the mediating effects of upward social comparison on social interaction of WeChat and rumination, and the chain mediating effect of upward social comparison on social interaction of WeChat to rumination were statistically significant among the influences of attitude toward social interaction of WeChat use on antenatal depression.

Table 4 shows the decomposition of direct and indirect effects of each factor in the structural model. The 95% CIs of the total and indirect effect value of attitude toward social interaction of WeChat use on antenatal depression did not contain 0, indicating that the two effects were statistically significant (total effect value=0.04; indirect effect value=0.09). The 95% CI of the direct effect value of attitude toward social interaction of WeChat use on antenatal depression included 0, indicating that this effect was not statistically significant. The mediating effect was composed of three indirect effects: path 1: attitude toward social interaction of WeChat use to upward social comparison on social interaction of WeChat to antenatal depression (indirect effect value=0.04); path 2: attitude toward social interaction of WeChat use to rumination to antenatal depression (indirect effect value=–0.02); and path 3: attitude toward social interaction of WeChat use to upward social comparison on social interaction of WeChat to rumination to antenatal depression (indirect effect value=0.07). All three indirect effects reached a significant level given that the 95% CIs did not contain 0. Comparing indirect effects 1, 2, and 3 showed that the bootstrap 95% CIs for the difference between indirect effects 1 and 2, indirect effects 1 and 3, and indirect effects 2 and 3 did not contain 0, indicating that there were statistically significant differences between them. Table 3 and Figure 2 show that upward social comparison on social interaction of WeChat and rumination played a statistically significant mediating role between attitude toward social interaction of WeChat use and antenatal depression.

**Table 3:** Regression analysis of the relationship among model variables (N=2661).

Variable			β^a^		*t* statistic (*df*)		*P* value		*R*			*R* ^2^			*F* statistic (*df*)	
**USCW^b^**										0.40			0.16			46.73 (11, 2649)
	Attitude to social interaction of WeChat use	.38		20.96 (2649)		<.001										
	Gestational age	.00		0.02 (2649)		.98										
	Education level	.06		2.98 (2649)		.003										
	Gravidity	–.01		-0.28 (2649)		.78										
	Parity	.01		0.32 (2649)		.75										
	Family income	–.01		–0.37 (2649)		.71										
	Quality of relationship with husband	.08		3.71 (2649)		<.001										
	Quality of relationship with mother-in-law	.04		1.97 (2649)		.049										
	Whether it is an unplanned pregnancy	.03		1.41 (2649)		.16										
	Living conditions	.06		3.09 (2649)		.002										
	Job	–.02		–0.90 (2649)		.37										
**Rumination**										0.46			0.21			58.58 (12, 2648)
	Attitude toward social interaction of WeChat use	–.05		–2.42 (2648)		.02										
	USCW	.38		20.02 (2648)		<.001										
	Gestational age	.00		0.24 (2648)		.81										
	Education level	.03		1.39 (2648)		.16										
	Gravidity	.02		0.86 (2648)		.39										
	Parity	-.03		-1.04 (2648)		.30										
	Family income	.03		1.67 (2648)		.09										
	Quality of relationship with husband	.09		4.09 (2648)		<.001										
	Quality of relationship with mother-in-law	.08		3.82 (2648)		<.001										
	Whether it is an unplanned pregnancy	–.03		–1.48 (2648)		.14										
	Living conditions	.12		6.20 (2648)		<.001										
	Job	.07		3.67 (2648)		<.001										
**Antenatal depression**										0.65			0.42			147.07 (13, 2647)
	Attitude toward social interaction of WeChat use	–.03		–1.94 (2647)		.05										
	USCW	.11		6.59 (2647)		<.001										
	Rumination	.50		30.29 (2647)		<.001										
	Gestational week	–.01		–0.92 (2647)		.36										
	Education level	–.10		–5.30 (2647)		<.001										
	Gravidity	.02		1.08 (2647)		.28										
	Parity	–.02		–0.83 (2647)		.41										
	Family income	–.05		–3.08 (2647)		.002										
	Quality of relationship with husband	.08		4.37 (2647)		<.001										
	Quality of relationship with mother-in-law	.08		4.63 (2647)		<.001										
	Whether it is an unplanned pregnancy	–.03		–2.13 (2647)		.03										
	Living conditions	.06		3.32 (2647)		<.001										
	Job	.03		1.56 (2647)		.12										

^a^Standardized coefficient.

^b^USCW: upward social comparison on social interaction of WeChat.

**Table 4:** Decomposition of effects of variables in structural equation modeling (N=2661).

Path			Effect		BootSE^a^		BootLLCI^b^		BootULCI^c^
Total effect			0.04		0.01		0.00		0.02
Direct effects			–0.02		0.01		–0.04		0.00
**Indirect effects**									
	Total indirect effects	0.09		0.01		0.07		0.12	
	Indirect effect 1^d^	0.04		0.01		0.03		0.06	
	Indirect effect 2^e^	–0.02		0.01		–0.04		–0.01	
	Indirect effect 3^f^	0.07		0.01		0.06		0.08	
	Indirect 1 versus Indirect 2	0.07		0.01		0.04		0.09	
	Indirect 1 versus Indirect 3	–0.03		0.01		–0.05		–0.01	
	Indirect 2 versus Indirect 3	–0.09		0.01		–0.12		–0.07	

^a^BootSE: bootstrap standard error.

^b^BootLLCI: bootstrap lower limit of confidence interval.

^c^BootULCI: bootstrap upper limit of confidence interval.

^d^Indirect effect path 1: attitude toward social interaction of WeChat use to upward social comparison on social interaction of WeChat to antenatal depression.

^e^Indirect effect path 2: attitude toward social interaction of WeChat use to rumination to antenatal depression.

^f^Indirect effect path 3: attitude toward social interaction of WeChat use to upward social comparison on social interaction of WeChat to rumination to antenatal depression.

## Discussion

### Principal Findings

#### Summary

This study established mediation models to analyze the relationship between social interaction of WeChat use intensity and antenatal depression. The results suggested that there are different relationships between the four aspects of WeChat use and antenatal depression. Attitude toward social interaction of WeChat use influenced antenatal depression through three paths: the mediating role of upward social comparison on social interaction of WeChat and rumination, and the chain mediating roles of upward social comparison on social interaction of WeChat and rumination. These findings reveal the complexities characterizing the relationships between WeChat use and antenatal depression.

#### Relationships Between Four Aspects of WeChat Use and Antenatal Depression

First, there was no relationship between antenatal depression and hours spent on WeChat use found in our study. Although recent meta-analyses consistently claim that there exist small, positive associations between hours spent on SNS and depression (*r*=0.11-0.13) [10], these effect sizes may not be adequate and reliable, likely because of the considerable and unexplained heterogeneity within the meta-analyses. In fact, time-based predictors may simply be too coarse and superficial to lead to meaningful associations between SNS use and depression. Thus, these analyses are not sufficient to capture individuals’ different motivations for using SNSs and experiences on SNSs, which impact individuals’ depression substantively. In addition, our result is consistent with the suggestions of converging reviews highlighting the need to establish nuanced content-based indicators, rather than performing additional studies on relationships between time spent using SNSs and depression.

Second, from a simple quantity-centric view, we found that having more WeChat friends was negatively associated with antenatal depression. This is likely because more SNS friends provides individuals with richer social capital benefits [44], stronger perceptions of social support [45,46], and the window into higher social status [47], which are all protective factors of depression according to previous systematic reviews or meta-analyses [48-50]. Furthermore, from an in-depth quality-centric perspective, we assume that various sources of SNS friends play different roles in individuals’ depression. In contrast to our finding, a recent meta-analysis suggested that there is no relation between Facebook friend counts and depression [51]. This difference is probably related to the fact that WeChat friends are mainly derived from people’s actual lives, whereas Facebook friends are often predominantly strangers, which indicates that only “actual” friends on SNSs can contribute to decreasing depression. Consistent with our speculation, a previous study showed that the total number of SNS friends alone does not predict bridging social capital, whereas the number of “actual” friends on the SNS does [44]. Further studies should confirm the characteristic effects of “actual” friends on SNSs upon individuals.

Finally, we found that antenatal depression was not related to the social interaction of WeChat use intensity, but was positively related to attitude toward social interaction of WeChat use. This seems to be a novel finding of our study, because previous studies on the association between intensity of SNS use and depression did not separately probe into the effect of attitude toward SNS. We can explain this finding from two perspectives. On the one hand, it may be that individuals with antenatal depression tend to score high on attitudinal questions [52,53]. For example, depressed individuals may be more dependent on WeChat interactions owing to the low social skills required in the offline context. On the other hand, it may also be that those who highly depend on WeChat subsequently develop antenatal depression. Some attitudinal questions (eg, I feel out of touch when I haven’t logged onto WeChat for a while) possess the characteristic of “WeChat addiction” [54]. Therefore, high scores on attitudinal questions represent a tendency for “WeChat addiction” to some extent, which is positively related to depression [55]. Due to the cross-sectional nature of the data, it is not possible to determine the directional causality of this association at present. Moreover, the positive correlation found between attitude toward SNS and depression is only based on Chinese pregnant women. Therefore, future studies are needed to explore the association and mechanisms among various populations through longitudinal or experimental designs. Nevertheless, this finding should be of interest and provide insight for clinical professionals and public health practitioners. It may be valuable to evaluate whether scoring high on SNS attitudinal questions is maladaptive, which may provide information to screen for antenatal depression or to disseminate targeted educational messages regarding antenatal depression.

The above distinct relations remind us that different aspects of SNS use should not be referred as “SNS use” generally. Only a fine-grained assessment can provide reliable information as to how individuals are influenced by SNSs.

#### Mediating Roles of Upward Social Comparison on Social Interaction of WeChat and Rumination

Previous research has shown that SNS use itself does not necessarily cause negative effects (see Table 5). Similar to these results, when we explored the mechanisms linking attitude toward social interaction of WeChat use and antenatal depression, we found that the concrete behaviors and feelings (such as upward social comparison on social interaction of WeChat and rumination) induced by WeChat use impact antenatal depression rather than the attitude toward social interaction of WeChat use itself. These findings are meaningful because they can remove the panic caused by the perception that SNS use on its own is harmful, and help to develop comprehensive and targeted interventions to reduce the harm caused by SNS use.

**Table 5:** Studies demonstrating that social networking site (SNS) use has no direct harmful effects.

Reference	Conclusion
Lin et al [56]	The problematic social media effects on psychological distress and mental quality of life were only indirect effects via generalized trust and perceived social support
Wang et al [57]	There is a significant mediating effect of SNS addiction between SNS use and mental health status, while the direct effect was insignificant
Niu et al [16]	Qzone use intensity is positively related to depression, and this relationship is fully mediated by negative social comparison on Qzone

In our study, attitude toward social interaction of WeChat use influenced antenatal depression through three paths: upward social comparison on social interaction of WeChat, rumination, and upward social comparison on social interaction of WeChat to rumination. First, attitude toward social interaction of WeChat use could increase risks of antenatal depression through increasing upward social comparison on social interaction of WeChat. This finding should come as no surprise as current evidence suggests that upward social comparison acts as a mediator between (subtypes of) SNS use and depression [18]. Our study is an expansion and enrichment to the research about upward social comparison on SNSs causing depression among pregnant women in mainland China.

Furthermore, after exposure to upward social comparison on social interaction of WeChat, rumination occurred, which could also increase risks of antenatal depression. This finding provides new insight to explain why upward social comparison on SNSs increases the risk of depression. Future research is needed to determine the characteristics of individuals that are most susceptible to rumination after upward social comparison on SNSs, which can help to implement preventive interventions targeted at this subgroup.

An intriguing finding was that attitude toward social interaction of WeChat use could decrease the risk of antenatal depression by decreasing rumination. Previous studies found that compared to general SNS use (such as time spent on SNS), high dependence on SNSs has the special potential to build and maintain multiform social capital [58]. Social capital can provide individuals with abundant resources such as emotional support and information. These resources not only provide distraction from ruminations but also encourage individuals to adopt effective problem-solving strategies to solve the stressor, thereby eliminating the trigger of rumination [59,60]. Therefore, attitude toward social interaction of WeChat use decreases an individual’s tendency to ruminate, likely through reaping social capital, and further decreases the risk of antenatal depression.

Compared to upward social comparison on social interaction of WeChat, rumination has stronger ability to increase levels of antenatal depression. This is probably because compared to upward social comparison on social interaction of WeChat, rumination is a more constant risk factor of depression. It is clear that rumination is the trigger and maintaining factor of depression [61]. However, upward social comparison on SNSs does not necessarily represent higher levels of depression, because it can sometimes be beneficial to individuals, such as by triggering inspiration and self-improvement [62-64]. Thus, we need to treat rumination with more caution than upward social comparison on social interaction of WeChat.

### Limitations

The cross-sectional nature of this study is a major limitation. Future studies should adopt longitudinal designs to explore potential causality. In addition, all of the questionnaires used in this study are based on self-reports. These results need to be verified and enriched by qualitative and/or experimental research to better understand how individuals are influenced by SNSs. Furthermore, this study only focused on the chain mediating roles of upward social comparison on social interaction of WeChat and rumination, omitting other meaningful variables, which need to be investigated through more studies. Finally, our study only demonstrates that upward social comparison on social interaction of WeChat and rumination can increase antenatal depression, whereas demonstrating the magnitude of these effects requires further research.

### Conclusions

In sum, our main goal is to explore the mediating role of upward social comparison on social interaction of WeChat and rumination between social interaction of WeChat use intensity and antenatal depression. The results of this study indicate that attitude toward social interaction of WeChat use (but not social interaction of WeChat use time and intensity) is associated with higher antenatal depression, which highlights that attitude toward SNS use should be valued and widely explored. Furthermore, the effect of attitude toward SNS use on antenatal depression depends on three pathways: the mediating roles of upward social comparison on social interaction of WeChat and rumination, and the chain mediating effect of upward social comparison on social interaction of WeChat and rumination. According to our results, intervention strategies focusing on lower upward social comparison and rumination may be helpful to reduce the risk of SNS use for depression. Moreover, it is assumed that SNS friends from an individual’s actual life (not the total number of SNS friends) are beneficial to decreasing depression. Although these findings need to be validated by longitudinal or experimental studies, they are helpful for researchers, health care professionals, and the public to better understand the inherent relationship between SNS use and antenatal depression.

## Appendix

Multimedia Appendix 1 Construct measurement.

Multimedia Appendix 2 STROBE (Strengthening The Reporting of Observational Studies in Epidemiology) Statement—Checklist of items that should be included in reports of cross-sectional studies.

## Abbreviations

EPDS: Edinburgh Postnatal Depression Scale
SNS: social networking site
STROBE: Strengthening The Reporting of Observational Studies in Epidemiology
