# Supplementary material for: Association Between Social Networking Site Use Intensity and Depression Among Chinese Pregnant Women: Cross-sectional Study
Source: J Med Internet Res. 2023 Mar 15;25:e41793. doi: 10.2196/41793 (PMC10132020; doi:10.2196/41793)
Supplement: Multimedia Appendix 1 [file jmir_v25i1e41793_app1.pdf]

Construct measurement

Construct		Item
Social Interaction of WeChat Use Intensity[a]	Hour Spent on Social Interaction of Wechat Use	About how many total WeChat friends do you have? (0=10 or less, 1=11-50, 2=51-100, 3=101-150, 4=151-200, 5 = 201-250, 6 = 251-300, 7=301–400, 8=more than 400)
	Number of WeChat Friends	In the past week, on average, approximately how many minutes per day have you spent on social interaction of WeChat Use? (0=less than 10, 1=10-30, 2=31-60, 3=1-2 hours, 4=2-3 hours, 5=more than 3 hours)
	Attitude to Social Interaction of WeChat use	Social interaction of WeChat use is part of my everyday activity
		I am proud to tell people I'm using social interaction of WeChat.
		Using social interaction of WeChat has become part of my daily routine
		I feel out of touch when I haven't logged onto social interaction of WeChat for a while
		I feel I am part of the social interaction of WeChat community
		I would be sorry if social interaction of WeChat shut down
Upward Social Comparison on Social Interaction of WeChat[a]	Item 1	I often consider my situation in life relative to that of other people on WeChat social interaction community.
	Item 2	If I want to find out how well I have done something, I compare what I have done with how others on WeChat social interaction community have done
	Item 3	When things get worse, I often think of those on WeChat social interaction community who are better than me
	Item 4	I often compare how I am doing socially (e.g., social skills, popularity) with other people on WeChat social interaction community
	Item 5	When considering whether I am capable of doing something, I often compare myself with others on WeChat social interaction community who perform better than me
	Item 6	I often compare myself with others with respect to what I have accomplished in life
Rumination[b,c]	Symptom-Based Rumination 1	Think about how alone you feel
	Symptom-Based	Think "I won't be able to do my job if I don't snap out

	Rumination 2	of this”
	Symptom-Based Rumination 3	Think about your feelings of fatigue and achiness
	Symptom-Based Rumination 4	Think about how hard it is to concentrate
	Brooding 1	Think “What am I doing to deserve this?”
	Symptom-Based Rumination 5	Think about how passive and unmotivated you feel
	Reflective Pondering 1	Analyze recent events to fry to understand why you are depressed
	Symptom-Based Rumination 6	Think about how you don’t seem to feel anything anymore
	Symptom-Based Rumination 7	Think “Why can’t I get going?”
	Brooding 2	Think “Why do I always react this way?”
	Reflective Pondering 2	Go away by yourself and think about why you feel this way
	Reflective Pondering 3	Write down what you are thinking about and analyze it
	Brooding 3	Think about a recent situation,wishing it had gone better
	Symptom-Based Rumination 8	Think “I won’t be able to concentrate if I keep feeling this way.”
	Brooding 4	Think “Why do I have problems other people don’t have?”
	Brooding 5	Think “Why can’t I handle things better?”
	Symptom-Based Rumination 9	Think about how sad you feel
	Symptom-Based Rumination 10	Think about all your shortcomings, failings, faults, mistakes
	Symptom-Based Rumination 11	Think about how you don’t feel up to doing anything
	Reflective Pondering 4	Analyze your personality to try to understand why you are depressed
	Reflective Pondering 5	Go someplace alone to think about your feelings
	Symptom-Based Rumination 12	Think about how angry you are with yourself
Antenatal Depression		In the past 7 days:
	Item 1[d]	I have been able to laugh and see the funny side of things (Not at all; Definitely not so much now; Not quite so much now; As much as I always could)

	Item 2[d]	I have looked forward with enjoyment to things (Hardly at all; Definitely less than I used to; Rather less than I used to; As much as I ever did)
	Item 3[e]	I have blamed myself unnecessarily when things went wrong (No, never; Not very often; Yes, some of the time; Yes, most of the time)
	Item 4[e]	I have been anxious or worried for no good reason (No, not at all; Hardly ever; Yes, sometimes; Yes, very often)
	Item 5[e]	I have felt scared or panicky for no very good reason (No, not at all; No, not much; Yes, sometimes; Yes, quite a lot)
	Item 6[e]	Things have been getting on top of me (No, I have been coping as well as ever; No, most of the time I have coped quite well; Yes, sometimes I haven't been coping as well as usual; Yes, most of the time I haven't been able to cope at all)
	Item 7[e]	I have been so unhappy that I have had difficulty Sleeping (No, not at all; Not very often; Yes, sometimes; Yes, most of the time)
	Item 8[e]	I have felt sad or miserable (No, not at all; Not very often; Yes, quite often; Yes, most of the time)
	Item 9[e]	I have been so unhappy that I have been crying (No, never; Only occasionally; Yes, quite often; Yes, most of the time)
	Item 10[e]	The thought of harming myself has occurred to me (Never; Hardly ever; Sometimes; Yes, quite often)

Notes. [a]Unless provided, response categories ranged from 1=strongly disagree to 5=strongly agree. [b]Items are numbered in the order they appear in the scale and are presented here in order of decreasing means. For the scale, items are preceded by the stem “When I am sad, down, or feel blue ⋯ .” [c]Response categories ranged from 1=not at all to 4=very often. [d]Response categories are scored 3, 2, 1, and 0. [e]Response categories are scored 0, 1, 2, and 3.
